# Quality of life outcomes in patients with localised renal cancer: a literature review

**DOI:** 10.1007/s00345-018-2415-3

**Published:** 2018-07-26

**Authors:** Sabrina H. Rossi, Tobias Klatte, Grant D. Stewart

**Affiliations:** 1 Academic Urology Group, University of Cambridge, Addenbrooke’s Hospital, Cambridge Biomedical Campus, Box 43, Hills Road, CB2 0QQ Cambridge, UK; 2 Department of Urology, Royal Bournemouth and Christchurch Hospitals, Bournemouth, UK

**Keywords:** Localised renal cell carcinoma, Quality of life, Utility, Nephrectomy, Review

## Abstract

**Purpose:**

Patients with localised renal cell carcinoma (RCC) can expect excellent oncologic outcomes. As such, there has been a shift towards maximising health-related quality of life (HRQoL). A greater understanding of HRQoL outcomes associated with different treatment options for RCC can facilitate patient-centred care, shared decision-making and enable cost utility analyses to guide health policies. The aim of this literature review was to evaluate the evidence regarding HRQoL following different management strategies for localised RCC.

**Methods:**

Three databases were searched to identify studies reporting HRQoL in patients with localised renal cancer, including Medline, the Tuft’s Medical Centre Cost Effectiveness Analysis registry and the EuroQol website.

**Results:**

Considerable methodological heterogeneity was noted. Laparoscopic nephrectomy was associated with significantly better short-term physical function compared to open surgery, although the effect on mental function was inconclusive. Nephron-sparing surgery was associated with better physical function compared to radical surgery. Patients’ perception of remaining renal function was a significant independent predictor of HRQoL, rather than surgery type. Tumour size, stage, post-operative complications, age, body mass index, occupational status, educational level and comorbidities were significant predictors of HRQoL. Only three studies were available regarding non-surgical management options and very little data were available regarding the impact of follow-up protocols and long-term effects of “cancer survivorship.”

**Conclusion:**

There is a need for validated and reproducible RCC-specific HRQoL instruments and standardisation amongst studies to enable comparisons. Increased awareness regarding determinants of poor HRQoL may enable high-risk patients to receive tailored support.

**Electronic supplementary material:**

The online version of this article (10.1007/s00345-018-2415-3) contains supplementary material, which is available to authorized users.

## Introduction

The incidence of renal cell carcinoma (RCC) is rising in Western Countries and is projected to rise further due to the aging population and rising prevalence of obesity [1]. As a result, a larger number of patients with RCC will require treatment annually, and live with the long-term sequelae of a cancer diagnosis. Due to the widespread use of abdominal imaging, a large proportion of individuals have small localised tumours, with excellent oncologic outcomes [2, 3]. As such, there has been a shift towards maximising health-related quality of life (HRQoL) and preserving renal function. Indeed, surveys of patients affected by RCC consistently highlight the need to place more emphasis on HRQoL, improved patient–doctor communication, patient information and education and support following a cancer diagnosis [4, 5]. A systematic review published in 2012 assessing quality of life (QoL) in patients undergoing management for localised RCC commented on the general lack of data, in particular concerning non-surgical management, with outcomes being inconsistently defined, measured and reported [6–8]. Since then, the urological community has acknowledged the importance of quantifying and maximising quality of life in patients with localised RCC, paralleled by an increasing in the number of publications regarding HRQoL and tools/questionnaires utilised for this purpose [9–11]. A greater understanding of HRQoL outcomes associated with different management options for RCC facilitates patient centred care and shared decision making. Additionally, there is an increasing emphasis on performing cost effectiveness analyses to quantify the incremental costs and quality adjusted life years of interventions in the diagnosis and management of RCC in the context of limited resources, to guide health policy.

This literature review describes key definitions and tools used to assess HRQoL outcomes in patients with localised RCC, as well as applications to health economic evaluation. In addition, we summarise the key evidence regarding HRQoL following available management options, including laparoscopic and open radical nephrectomy (LRN and ORN), laparoscopic and open partial nephrectomy (LPN and OPN), ablation and active surveillance (AS).

## Methods

The Medline database was searched to identify quality of life studies for localised RCC (updated to March 2018). The following keywords and medical subject headings were utilised: quality of life, EuroQol, nephrectomy, renal cell carcinoma, kidney cancer, renal mass, renal carcinoma and renal cancer. The search strategy was limited to English language studies. Where reviews were identified, the reference list was manually searched by the study authors. Furthermore, as quality of life studies are often utilised in health economic evaluation, the Tuft’s Medical Centre Cost Effectiveness Analysis registry was searched to identify studies reporting QoL in patients with localised renal cancer. Lastly, the EuroQol website was searched to identify studies using the EQ-5D by searching for any publications containing the key words “kidney” or “renal”, as this is the preferred questionnaire to assess utilities by the National Institute for Health and Care Excellence (NICE) [12].

## Results

The search identified 31 studies within the Tuft’s Medical Centre Cost Effectiveness Analysis registry. The EQ-5D website revealed 164 studies containing the key words “kidney” or “renal”; however, only one was pertinent to renal cancer. Overall, including the Medline search, a total of 61 full texts were reviewed. A number of methodological considerations were noted regarding the studies identified by this review. A number of different quality of life questionnaires and outcomes were reported, and there was a wide variation in the time point assessed (ranging from a few weeks to many years following management of renal cancer). Only one randomised control trial was identified, some studies were prospective in nature although the majority were retrospective. Most studies contained a small sample size including a heterogeneous study population and low response rates and were underpowered to detect differences in QoL between interventions and to assess determinants of QoL. Furthermore, a number of studies did not assess QoL at baseline prior to intervention, therefore, reducing the meaningfulness of comparisons between intervention types. As a result, there is a large degree of heterogeneity amongst studies, which limits our ability to pool and directly compare data.

### Definitions and instruments

HRQoL is a multidimensional concept which is difficult to characterise and has, therefore, been associated with a myriad of varying definitions in the medical literature [13, 14]. Although QoL and HRQoL are often used interchangeably in the literature, HRQoL is specific to the patient’s perception of the disease, including the diagnosis, symptoms, treatment and prognosis [13, 14]. This encompasses physical, emotional, cognitive and social components, including the individual’s functioning and wellbeing. QoL is a broader concept and is affected not only by health, but by other domains such as housing, employment, safety and freedom [15]. Both QoL and HRQoL are subjective measures which can be assessed in a validated and reproducible way through questionnaires of patient reported outcome measures (PROMs). PROMs are defined as measures which are directly reported by patients, without interpretation by a health care professional and refer to the method in which HRQoL is assessed rather than the content itself [15].

A number of generic and cancer-specific instruments have been developed to assess health-related QoL in patients with cancer and more specifically, patients with RCC (Table 1). Disease-specific questionnaires for patients with localised disease assess symptoms such as flank pain and haematuria, as well as more general cancer symptoms such as weight loss and fatigue [6]. Comparative studies evaluating different RCC-specific questionnaires have demonstrated that generally very similar symptoms are assessed, with the main differences being questionnaire phrasing, length and ease of use [16]. Questionnaires assess different domains of QoL and there is a fine balance between collecting adequate data regarding all domains and maintaining the instrument brief and practical.

**Table 1:** Generic and cancer-specific instruments which have been utilised to assess health-related quality of life in renal cancer

Instrument	Description
Generic instruments assessing quality of life	
RAND medical outcome survey Short Form 36 (SF-36) and Short Form 12 (SF-12) [52]	SF-36 is a questionnaire with 36 multiple choice items. Eight domains are assessed and two summary scores are produced (physical and mental component summary scores). The eight domains are: physical function, role limitations due to physical health problems, bodily pain, general health perception, emotional wellbeing, role limitations due to emotional problems, energy/fatigue, and social function. A shortened version has been developed, the SF-12, which can be completed in a third of the time as it only contains 12 items. Results from the SF-36 and SF-12 can be classified according to the SF-6D, which allows preference based health state utilities to be derived for health economic evaluation
EuroQol (EQ-5D) [53]	The EuroQol questionnaire contains five dimensions: mobility, self-care, usual activities, pain/discomfort and anxiety/depression. For adults, two versions exist; the EQ-5D-5L which contains five levels for each of the five domains and the EQ-5D-3L which contains three levels. According to NICE guidelines, the recommended method to assess utilities is using the EQ-5D questionnaire [12]
Convalescence and Recovery Evaluation (CARE) [54]	This questionnaire was designed to assess short term physical and cognitive function following abdominopelvic surgery. It contains 27 items and four domains: pain, gastrointestinal, cognitive, and activity
Cancer-specific instruments assessing quality of life	
Cancer Rehabilitation Evaluation System-Short Form (CARES-SF) [55]	Cancer-specific quality of life questionnaire containing five domains: physical, psychosocial, medical, sexual functioning and marital
European Organization for Research and Treatment of Cancer (EORTC) Quality of Life Questionnaire (QLQ) C30 [56]	General quality of life questionnaire used in different cancer types designed to assess QoL in clinical oncology trials. The questionnaire assesses global QoL, symptom scales and five functional scales (physical, role, cognitive, emotional, and social). A renal cancer-specific domain has recently been developed, although it remains to be externally validated [6]. This includes the following disease-specific items: flank pain, oedema, haematuria and urinary tract infections [6]
Functional Assessment of Cancer Therapy-General (FACT-G) [57]	The FACT-G questionnaire contains core domains and can be used in all cancer types, including renal cancer. The following domains are assessed: physical wellbeing, social/family wellbeing, emotional wellbeing, and functional wellbeing. A number of disease-specific modifications exist, which are pertinent to advanced RCC (see below)
Functional Assessment of Cancer Therapy-Kidney Symptom Index (FKSI) [58, 59]	The FKSI is used in individuals with advanced renal cancer to assess disease-related symptoms, treatment side effects, and general function and wellbeing. A number of variations/subsets have been published, each containing a different number of items. The FKSI-15 contains 15 items, the FSKI-19 contains 19 items, while the FKSI-Disease Related Symptoms (FKSI-DRS) focuses specifically on disease-related, rather than treatment-related quality of life. For example the FKSI-DRS assesses haematuria, fevers, chest symptoms, bone pain and fatigue
Renal Cell Carcinoma-Symptom Index (RCC-SI) [60]	This questionnaire assesses renal cancer-specific physical and psychological symptoms and can be used in both localised and advanced renal cancer. Items assessed include: haematuria, difficulty passing urine, pain, chest and bowel symptoms, sleep and fatigue
Instruments to assess psychological wellbeing	
Impact of Events Scale (IES) [61]	Questionnaire to assess distress by evaluating two domains: intrusive thoughts and avoidance behaviour
Hospital Anxiety and Depression Scale (HADS) [62]	This questionnaire contains 14 multiple choice questions assessing anxiety and depression
Mishel Uncertainty in Illness Scale (MUIS) [63]	This consists of four domains regarding diagnosis, treatment, disease severity and outcomes: ambiguity, complexity, lack of information and unpredictability

### Surgical management

#### Laparoscopic versus open surgery

It is widely recognised that the laparoscopic approach has a significant impact on reducing patients’ hospital stay and return to daily activities [17]. A number of studies have evaluated whether this translates into a measurable quality of life benefit (Table 2). Burgess et al. performed a randomized control trial of laparoscopic versus open nephrectomy. The laparoscopic approach was associated with significantly reduced post-operative visual analogue pain scores and faster return to normal daily activities (42 vs 62 days; *p* = 0.04). However, no significant difference was noted between EQ-5D scores in the two groups at 3 and 12 months post-operatively [18]. Parker et al. demonstrated that patients undergoing laparoscopic renal surgery had significantly better physical component scores (PCS) on SF-36 questionnaires compared to those undergoing open surgery, and return to baseline was quicker. However, although benefits on physical components were evident in the short term for patients undergoing LRN, the differences between laparoscopic and open surgery disappeared after a few months. Additionally, mental component scores (MCS) on SF-36 and Impact of Event Scale scores (IES), assessing intrusive thoughts and avoidance behaviour, were not significantly different [19]. Similarly, Acar et al. demonstrated shorter hospital stay, earlier ambulation and better general health perception following laparoscopic surgery. Following both laparoscopic and open surgery, by 6 months physical function scores were improved and were better than baseline [20]. Additionally, Harryman et al. suggested that the laparoscopic approach was associated not only with higher physical but also a significantly higher mental component score on SF-36 in the short-term post-operative period [21].

**Table 2:** Summary of key studies evaluating health-related quality of life in individuals with localised renal cancer undergoing surgical management

Study	Sample size (N)	Surgery type	Timing of QoL assessment	Questionnaire	Key findings
Laparoscopic vs open surgery					
Burgess et al. [18]	*N* = 45	LRN vs ORN	Baseline, 3 and 12 months	EQ-5D VAS pain score	LRN: significantly reduced post-operative pain and faster return to normal daily activities EQ-5D data was not significant therefore not reported
Harryman et al. [21]	*N* = 100 Only 71 patients completed all assessments	LN vs ON	Baseline, 2 days and 1 month	SF-36 VAS pain score	LN: significantly better PCS, MCS and reduced pain
Kim et al. [8]	*N* = 71 Only 60 patients completed questionnaire at baseline and at least one other time point	ON, LN and robotic assisted nephrectomy	Baseline, 2, 4, 12, and 24 weeks	CARE SF-12	Study not powered to allow comparisons amongst patients undergoing different surgical management options
Parker et al. [19]	*N* = 172 Only 64 patients completed all assessments	LRN, LPN, ORN, OPN	Baseline, 3 weeks, 2, 3, 6, and 12 months	SF-36 CARES-SF IES Fear of recurrence	LN vs ON: LN associated with significantly better PCS, quicker return to baseline, but short term benefit only RN vs PN: RN associated with significantly better cancer-specific QoL No difference in MCS, IES and fear of recurrence between surgery types Post-operative GFR was significantly associated with PCS, IES and fear of recurrence
Acar et al. [20]	*N* = 72	LRN vs ORN	Baseline, 1 and 6 months	SF-36 VAS pain score	LRN: shorter hospital stay, earlier ambulation and better general health perception VAS pain scores were similar
Becker et al. [28]	*N* = 110	LPN vs OPN	Median: 49 months	EORTC-QLQ-C30	No significant difference in post-operative HRQoL in the two groups
Nephron-sparing vs radical surgery					
Clark et al. [22]	*N* = 97	PN vs RN	NR	SF-36 IES-R Fear of recurrence	Patients’ perception of remaining renal function, rather than surgery type, was a significant and independent predictor HRQoL Self-reporting less renal function: worse PCS and IES, more negative thoughts regarding cancer recurrence and reduced renal function
Shinohara et al. [26]	*N* = 50	PN vs RN		EORTC-QLQ-C30	PN: significantly better physical function, less pain, fatigue, sleep disturbance and constipation
Ficarra et al. [23]	*N* = 144	PN vs RN	Median: 55 ± 36 months	GHQ HADS SPQ List of threatening experiences	PN: significantly lower levels of anxiety and depression No significant difference in other domains
Poulakis et al. [24]	*N* = 357	PN vs RN	NR	SF-36 EORTC-QLQ-C30 IES-R Fear of recurrence Worry about having fewer than two normal kidneys	PN: significantly better physical function, role functioning and less pain and fatigue No difference in IES and fear of recurrence
Gratzke et al. [27]	*N* = 117 Only 84 patients completed assessments	RRN vs ORN vs OPN	Mean: 22 months	SF-36	No significant difference between HRQoL in the three surgical groups
Novara et al. [29]	*N* = 129	PN vs RN	Baseline, 6 and 12 months	SF-36	Type of surgery did not affect return to baseline Following operative management for RCC, physical function may be worse than the reference population due to the insult of surgery, but emotional and psychological wellbeing is higher as the tumour has been removed
Azawi et al. [25]	*N* = 152	PN vs RN	Three cohorts: < 2 years from surgery, 2–4 and > 4 years	EORTC-QLQ-C30 RQRC	PN: significantly better global QoL, physical function, role functioning and less fatigue

*CARES-SF* Cancer Rehabilitation Evaluation System-Short Form, *EORTC-QLQ-C30* European Organization for Research and Treatment of Cancer Quality of Life Questionnaire C30, *GHQ* General Health Questionnaire, *HADS* Hospital Anxiety and Depression Scale, *IES-R* Impact of Events Scale Revised, *LPN* laparoscopic partial nephrectomy; *LRN* laparoscopic radical nephrectomy, *MCS* mental component score on SF-36, *NR* Not reported, *OPN* open partial nephrectomy, *ORN* open radical nephrectomy, *PCS* physical component score on SF-36, *QoL* Quality of life; *RQRC* Rehabilitation Questionnaire for Renal Cancer, *RRN* retroperitoneoscopic radical nephrectomy, *SF-12* Short Form 12, *SF-36* Short Form 36, *SPQ* Social Problem Questionnaire, *VAS* Visual Analogue Scale

#### Nephron-sparing vs radical surgery

The impact of nephron-sparing surgery (NSS) compared to radical nephrectomy (RN) on HRQoL scores is multilayered and remains somewhat unclear. The aim of NSS is to preserve renal function and several studies suggest that preserved glomerular filtration rate is associated with measurable benefits in patient-reported HRQoL (Table 2). Clark et al. evaluated individuals with localised renal tumours undergoing RN and PN. Patients’ perception of remaining renal function was a significant and independent predictor of HRQoL, rather than surgery type. Patients who self-reported as having less remaining renal parenchyma had significantly worse physical health on SF-36, higher intrusion and avoidance scores on IES, and more negative thoughts regarding cancer recurrence or reduced renal function [22]. Several studies demonstrated similar findings: patients undergoing RN had more worry regarding potential loss or damage to the single functioning contralateral kidney [19, 23]. Furthermore, several studies demonstrate that PN is associated with significantly better physical function scores compared to RN, as well as reduced symptoms such as fatigue, sleep disturbance and pain [24–26]. Ficarra et al. report that levels of anxiety and depression in patients undergoing RN and PN are very low, however significantly lower in the PN group [23]. However, some inconsistencies remain. Parker et al. demonstrated better cancer-specific QoL in patients who underwent RN compared to PN [19]. In addition, a number of studies have failed to show a significant difference in quality of life following RN versus PN, though this may be related to low sample size or the timing of the assessment in relation to surgery [27].

#### Recovery following surgical management

A number of studies suggest that HRQoL returns to baseline following surgical management of localised RCC and evaluate temporal trends (Table 2). Gratzke et al. assessed HRQoL using the SF-36 questionnaire in patients with stage I and stage II RCC following retroperitoneoscopic radical nephrectomy, open RN and open PN. There was no significant difference between HRQoL in the three surgical groups and physical condition scores in RCC patients 1 year following surgery were actually higher than an age- and sex-matched reference population [27]. This may be because operative candidates are more likely to be fitter and less comorbid than age- and sex-matched controls from the general population. Similarly, Becker et al. demonstrated that there was no significant difference in post-operative HRQoL in patients with stage I disease undergoing laparoscopic or open PN. Renal cancer patients’ HRQoL scores were either better or the same for males and females, respectively, compared to a German reference population [28]. Kim et al. evaluated the Convalescence and Recovery Evaluation (CARE) and SF-12 preoperatively and following open, laparoscopic and robotic renal surgery (2, 4, 12, and 24 weeks post-operatively). A temporal trend was observed in HRQoL outcomes: over half of patients returned to baseline by 4 weeks and over 80% of patients returned to baseline HRQoL by 12 weeks post-operatively. Unfortunately, the study was underpowered and did not allow comparisons amongst patients undergoing different surgical management [8]. Novara et al. compared SF-36 scores in patients undergoing NSS or RN. Baseline pre-operative scores were not significantly different between individuals with RCC and the age- and sex-matched reference population. The study showed that approximately 50–80% of patients returned to baseline at 24 and 52 weeks post-operatively. Furthermore, it was suggested that following operative management for RCC, physical function may be worse than the reference population due to the insult of surgery, but emotional and psychological wellbeing is higher as the tumour has been removed [29]. In contrast, Parker et al. suggest that following laparoscopic surgery, the observed reduction in physical scores on HRQoL improve by 3 months; however, anxiety and worry regarding cancer recurrence remain long term [19].

#### Determinants of post-operative HRQoL

Identifying determinants of poor HRQoL outcomes is crucial to enable the identification of high-risk groups, which would benefit from additional psychological and physical support. Unsurprisingly, patients with recurrence have significantly worse HRQoL. Furthermore, individuals with more advanced tumour size and pathological stage experience significantly greater fear of recurrence, intrusive thoughts, hyperarousal and avoidance behaviour [24]. Patients with a low pre-operative renal function or single kidney undergoing “imperative” NSS have significantly increased worry regarding cancer recurrence and worsening renal function, compared to individuals undergoing “elective” NSS [22, 24]. Individuals undergoing “imperative” NSS are also significantly less likely to return to baseline social and physical function on SF-36 scores post-operatively compared to individuals undergoing “elective” surgery [29].

Complications following renal surgery are a major determinant of HRQoL [27]. This is a well-documented effect, and a recently performed meta-analysis demonstrated that across all surgical specialities, post-operative complications are associated with decreased physical, social and emotional wellbeing. Most importantly, there was a discrepancy between the reported severity of complications by health care professionals and patients, meaning that even what may be perceived as mild complications can have a profound impact on the patients’ recovery [30]. Age is an established determinant of QoL following operative management. Increasing age is associated with longer time to return to baseline physical function, higher fear of recurrence but less intrusive thoughts and avoidance behaviour [19, 29]. As expected, body mass index, occupational status, educational level and comorbidities were significantly associated with return to baseline QoL assessments on the SF-36 at 6 months and 1 year following operative management [29].

### Non-surgical management

Data regarding the quality of life of patients undergoing non-surgical management are sparse [31]. Onishi et al. performed a small trial (*n* = 37) comparing QoL using SF-36 questionnaire in patients undergoing percutaneous radiofrequency ablation (RFA) and LRN [32]. Patients undergoing RFA were significantly older and had significantly worse baseline HRQoL compared to individuals undergoing LRN, reflecting inherent differences in the two populations. As expected, LRN was associated with a post-operative HRQoL decrement in physical functioning, role-physical functioning and role-emotional functioning, which resolved by 4–11 weeks. Patients undergoing RFA did not experience a decrement in HRQoL following the procedure and on the contrary, perceived a steady increase in HRQoL in role-physical functioning, vitality and mental health. RFA remains an excellent alternative for patients with small renal masses who opt not to undergo NSS due to comorbidities or patient choice. However, more data are required to evaluate the long-term impact of tumour recurrence and the need for repeat RFA interventions on patients’ HRQoL, in a larger patient cohort.

Parker et al. evaluated HRQoL in 100 patients with clinically T1 and T2 renal masses undergoing active surveillance over a 24-month period. As time progressed, there was a significant worsening in the physical component of the SF-36, but a reduction in intrusive thoughts on IES. Illness uncertainty was a significant predictor of overall and cancer-specific HRQoL scores and HRQoL was significantly predicted by age, gender, renal function, tumour size and comorbidities [33]. Unfortunately, the study did not compare HRQoL in patients undergoing different treatment options. More recently, Patel et al. evaluated 539 patients with small renal masses (clinically T1a) enrolled in the prospective, multi-centre Delayed Intervention and Surveillance for Small Renal Masses (DISSRM) Registry [34]. Patients were offered the choice between AS and primary treatment (PT). Patients undergoing AS were nearly 10 years older and had more comorbidities at baseline than patients choosing PT. Over the observation period, PCS scores on SF-12 worsened similarly with time in both PI and AS groups; however, PCS remained significantly worse in the AS group compared to PT group at all time points, reflecting the initial poorer overall health and comorbidities. Comorbidities, as measured by Eastern Cooperative Oncology Group performance status and cardiovascular index, were significant predictors of HRQoL. Most importantly, AS (and crossing over from AS to treatment) was not associated with a reduction in MCS scores compared to PT. In itself, shared decision making between patients with cancer and health care professionals regarding treatment choices has been shown to increase HRQoL [35]. Similarly, studies in patients undergoing AS or PT for prostate cancer have also failed to show a significant association between AS and reduced HRQoL [36].

### Follow-up and surveillance

The optimal evidence-based follow-up protocol for patients with localised RCC who have undergone curative treatment has yet to be defined. Follow-up recommendations from different urological societies are conflicting; however, broadly it is recommended for patients to undergo annual abdominal ± chest imaging for several years based on prognostic risk [37]. Patients may experience increased anxiety in relation to continued investigations, especially in the context of time delays between undergoing imaging and receiving results. A recently performed systematic review of studies assessing HRQoL in patients with colorectal cancer undergoing regular follow-up suggested that over a third of studies reported negative perceptions of follow-up, including increased anxiety and stress [38]. There is a lack of data regarding the impact of follow-up protocols on HRQoL and patient preferences in RCC.

Kent et al. assessed long-term HRQoL in patients defined as “cancer survivors.” The mean interval after RCC diagnosis was 77 months. Although the stage of the disease was not reported, patients with RCC had significantly worse PCS but similar MCS compared to individuals from the general population. Overall, scores for RCC survivors were similar to that of other cancers [39]. Sexual function may play an important role in patients’ QoL; however, this domain is often overlooked by health care professionals. There has been some suggestion that RCC survivors may have relatively poor sexual functioning compared to breast cancer survivors [40]. More research is required in this field [5].

### HRQoL and cost utility analyses

HRQoL has also arisen as a key component of cost utility analyses and economic evaluation. In the context of a finite healthcare budget, decisions need to be made regarding which interventions can be preferentially funded; thus, there is a drive to assess not only effectiveness but also cost effectiveness of interventions [41]. Cost utility analyses evaluate costs and quality adjusted life years (QALYs), a measure of length and quality of life, enabling a range of interventions to be compared against each other [41]. A large number of cost utility analyses have been undertaken to evaluate systemic therapies in metastatic RCC and more recently there has been a rise in the number of analyses performed to evaluate management options in localised disease [42–47]. An understanding of how utilities are derived highlights key challenges in this field. Utilities consist of preference-based values that are applied to health states [48]. Perfect health is associated with a utility of 1 and death of 0, although some conditions may be worse than death and, therefore, associated with a negative value. More desirable or preferable utilities are associated with higher values on this scale. To derive utilities, patients with the condition of interest are asked to fill in HRQoL questionnaires, such as the EQ-5D, SF-36 or EORTC-QLQ-C30. Individuals from the general public are subsequently asked to assign relative values or preferences to each of the health states reported by the patients, based on methods such as standard gamble, time trade-off, or Visual Analogue Scale [49]. Clearly, the results of a cost utility analysis are dependent on the accuracy of the HRQoL studies and country-specific value sets used to determine the utilities. Due to the lack of existing evidence, earlier cost effectiveness analyses estimated health state utilities for localised RCC based on clinical expert judgment and data from other disease areas, such as colorectal cancer [42, 43, 46, 47]. More recently, Chang et al. estimated a health state utility of 0.7 for OPN and 0.88 for LPN relative to perfect health; however, the study did not describe the methods used to derive these values [44]. Klinghoffer et al. calculated health state utilities for individuals undergoing LRN and OPN based on the results of SF-36 questionnaire mapped to utility values (utility for LRN: 0.73; utility for OPN: 0.744) [45]. However, utilities were estimated for LPN as no data were available, and robotic and ORN surgery were not included in the analysis. This highlights a lack of data regarding utilities for localised RCC and demonstrates a clear priority for further research.

### Critical review and future directions

Despite increases in the number of studies reporting HRQoL in localised RCC in recent years, a number of methodological considerations remain. A variety of different questionnaires are used, limiting the ability to compare results across studies. Although the majority of studies utilise the SF-36, it has been postulated that such generic questionnaires do not accurately capture RCC-specific QoL [6]. More recently, there has been a drive to develop RCC-specific HRQoL questionnaires, aimed at patients with localised disease; however, these are not yet used routinely in clinical studies [6]. There is a need for validated and reproducible renal cancer-specific HRQoL instruments, and standardisation amongst studies to enable comparisons [50].

A number of areas were identified in which further HRQoL research is necessary. Notably, there was a lack of data regarding robotic surgery, as studies were underpowered to report on this, as well as ablation and AS. The European Active SurveillancE of Renal cancer (EASE) study is currently underway and it will provide crucial information regarding HRQoL in patients undergoing AS, as measured by EORTC QLQ-C30 and HADS questionnaires [51]. Future studies assessing management strategies for localised RCC should routinely report PROMs. More research is also required regarding the long-term impact of “cancer survivorship” in patients with RCC, including the impact on sexual function [5]. We identified only two studies reporting utilities in localised RCC based on HRQoL, and methods were incompletely reported. There is, therefore, a need for transparent studies evaluating HRQoL and mapping these to health state utility values to enable cost effectiveness analyses in localised RCC.

## Conclusion

In summary, laparoscopic nephrectomy was associated with significantly better short-term physical function compared to open surgery, although the effect on mental function was inconclusive. The effect of NSS on HRQoL was less clear cut. Overall, it was suggested that NSS was associated with better physical function as well as reduced intrusive thoughts, avoidance behaviour, anxiety and worry. Ablative therapy and AS were not associated with worse psychological outcomes compared to operative management, though data were sparse.

This review highlights the importance of assessing HRQoL in patients undergoing management for localised RCC. This may enhance patient centred care and shared decision making as well as enabling more accurate health economic evaluations to guide health policy. Further education is required amongst renal cancer surgeons to increase awareness regarding determinants of poor HRQoL following management for RCC. This will enable patients at high risk of worse HRQoL to be identified and offered tailored support, including psychological interventions and increased education. Further education may also help us to overcome the well-documented discrepancies between health care professionals and patient perception of the cancer consultation [5].

## Electronic supplementary material

Below is the link to the electronic supplementary material.

Supplementary material 1 (DOCX 27 kb)

## Abbreviations

AS: Active surveillance
CARE: Convalescence and Recovery Evaluation
CARES-SF: Cancer Rehabilitation Evaluation System-Short Form
DISSRM: Delayed Intervention and Surveillance for Small Renal Masses
EASE: European Active SurveillancE of renal cell carcinoma study
EORTC-QLQ-C30: European Organization for Research and Treatment of Cancer-Quality of Life Questionnaire-C30
EQ-5D: EuroQol questionnaire
FACT-G: Functional Assessment of Cancer Therapy-General
FKSI: Functional Assessment of Cancer Therapy-Kidney Symptom Index
GHQ: General Health Questionnaire
HADS: Hospital Anxiety and Depression Scale
HRQoL: Health-related quality of life
IES-R: Impact of Events Scale-Revised
LPN: Laparoscopic partial nephrectomy
LRN: Laparoscopic radical nephrectomy
MCS: Mental component score on SF-36
MUIS: Mishel Uncertainty in Illness Scale
NICE: National Institute for Health and Care Excellence
NR: Not reported
NSS: Nephron-sparing surgery
OPN: Open partial nephrectomy
ORN: Open radical nephrectomy
PCS: Physical component score on SF-36
PROMs: Patient reported outcome measures
PT: Primary treatment
QoL: Quality of life
RCC: Renal cell carcinoma
RCC-SI: Renal Cell Carcinoma-Symptom Index
RQRC: Rehabilitation Questionnaire for Renal Cancer
RRN: Retroperitoneoscopic radical nephrectomy
SF-12: Short Form 12
SF-36: Short Form 36
SPQ: Social Problem Questionnaire
VAS: Visual Analogue Scale
