# Supplementary material for: Quality of life outcomes in patients with localised renal cancer: a literature review
Source: World J Urol. 2018 Jul 26;36(12):1961–72. doi: 10.1007/s00345-018-2415-3 (PMC6280814; doi:10.1007/s00345-018-2415-3)
Supplement: Supplementary file 1 — Supplementary material 1 (DOCX 27 kb) [file 345_2018_2415_MOESM1_ESM.docx]

**SUPPLEMENTAL DATA**

Table S1 lists generic and cancer specific instruments which have been utilised to assess health related quality of life in renal cancer. Details are provided regarding how to access each individual instrument, as well as scoring and interpretation manuals.

Table S1:

| **Generic instruments assessing quality of life** | |
| --- | --- |
| *Instrument* | *Access details* |
| RAND Medical Outcome Survey Short Form 36 (SF-36) and Short Form 12 (SF-12) [1] | The SF-36 is available on the following website: https://www.rand.org/health/surveys_tools/mos/36-item-short-form/survey-instrument.html |
| Euroqol (EQ-5D) [2] | Access to the EQ-5D is granted on permission (licensing fees may apply). Users are required to register their study/project using a registration form on the Euroqol website.  Website: https://euroqol.org/support/how-to-obtain-eq-5d/ |
| Convalescence and  recovery evaluation (CARE) [3] | This instrument and details regarding scoring are available in a publication by Hollenbeck *et al* [3]. |
| **Cancer specific instruments assessing quality of life** | |
| *Instrument* | *Access details* |
| Cancer Rehabilitation  Evaluation System- Short Form (CARES-SF) [4] | The following website contains both the standard and short form versions of the CARES questionnaire, along with user manuals:  https://cancer.ucla.edu/patient-care/survivorship/for-healthcare-providers/cancer-rehabilitation-evaluation-system-cares |
| European Organization for Research and Treatment of  Cancer (EORTC) Quality of Life Questionnaire (QLQ) C30 [5] [6] | This is a copyrighted instrument, which is available on request (fees may apply) via the following website: http://groups.eortc.be/qol/eortc-qlq-c30 |
| Functional Assessment of Cancer Therapy – General (FACT-G) [7] | This is a copyrighted instrument. Access to scoring and interpretation manuals is available on request (fees may apply) via the following website:  http://www.facit.org/facitorg/questionnaires |
| Functional Assessment of Cancer Therapy-Kidney Symptom Index (FKSI) [8,9] | This is a copyrighted instrument. Access to scoring and interpretation manuals is available on request (fees may apply) via the following website:  http://www.facit.org/facitorg/questionnaires |
| Renal cell carcinoma symptom index (RCC-SI) [10] | This instrument and details regarding scoring are available in a publication by Harding *et al* [10]. |

**References**

1. Ware JE, Jr., Sherbourne CD (1992) The MOS 36-item short-form health survey (SF-36). I. Conceptual framework and item selection. Medical care 30 (6):473-483

2. Brooks RG, Jendteg S, Lindgren B, Persson U, Bjork S (1991) EuroQol: health-related quality of life measurement. Results of the Swedish questionnaire exercise. Health Policy 18 (1):37-48

3. Hollenbeck BK, Dunn RL, Wolf JS, Jr., Sanda MG, Wood DP, Gilbert SM, Weizer AZ, Montie JE, Wei JT (2008) Development and validation of the convalescence and recovery evaluation (CARE) for measuring quality of life after surgery. Qual Life Res 17 (6):915-926. doi:10.1007/s11136-008-9366-x

4. Schag CA, Ganz PA, Heinrich RL (1991) CAncer Rehabilitation Evaluation System--short form (CARES-SF). A cancer specific rehabilitation and quality of life instrument. Cancer 68 (6):1406-1413

5. Aaronson NK, Ahmedzai S, Bergman B, Bullinger M, Cull A, Duez NJ, Filiberti A, Flechtner H, Fleishman SB, de Haes JC, et al. (1993) The European Organization for Research and Treatment of Cancer QLQ-C30: a quality-of-life instrument for use in international clinical trials in oncology. J Natl Cancer Inst 85 (5):365-376

6. Beisland E, Aarstad HJ, Aarstad AK, Bakke A, Bostad L, Beisland C (2016) Development of a disease-specific health-related quality of life (HRQoL) questionnaire intended to be used in conjunction with the general European Organization for Research and Treatment of Cancer (EORTC) Quality of Life Questionnaire (QLQ) in renal cell carcinoma patients. Acta Oncol 55 (3):349-356. doi:10.3109/0284186X.2015.1063776

7. Cella DF, Tulsky DS, Gray G, Sarafian B, Linn E, Bonomi A, Silberman M, Yellen SB, Winicour P, Brannon J, et al. (1993) The Functional Assessment of Cancer Therapy scale: development and validation of the general measure. J Clin Oncol 11 (3):570-579

8. Rothrock NE, Jensen SE, Beaumont JL, Abernethy AP, Jacobsen PB, Syrjala K, Cella D (2013) Development and Initial Validation of the NCCN/FACT Symptom Index for Advanced Kidney Cancer. Value Health 16 (5):789-796. doi:10.1016/j.jval.2013.04.015

9. Cella D, Yount S, Brucker PS, Du H, Bukowski R, Vogelzang N, Bro WP (2007) Development and validation of a scale to measure disease-related symptoms of kidney cancer. Value Health 10 (4):285-293. doi:10.1111/j.1524-4733.2007.00183.x

10. Harding G, Cella D, Robinson D, Jr., Mahadevia PJ, Clark J, Revicki DA (2007) Symptom burden among patients with renal cell carcinoma (RCC): content for a symptom index. Health Qual Life Outcomes 5:34. doi:10.1186/1477-7525-5-34

11. Horowitz M, Wilner N, Alvarez W (1979) Impact of Event Scale: a measure of subjective stress. Psychosom Med 41 (3):209-218

12. Zigmond AS, Snaith RP (1983) The hospital anxiety and depression scale. Acta Psychiatr Scand 67 (6):361-370

13. Mishel MH (1981) The measurement of uncertainty in illness. Nurs Res 30 (5):258-263
